# Isolated growth hormone deficiency type IA due to a novel *GH1* variant: a case report

**DOI:** 10.1186/s12920-021-01057-z

**Published:** 2021-09-02

**Authors:** Xi Yang, Mingming Yuan, Zhuoguang Li, Yanqin Ying, Ling Hou, Xiaoping Luo

**Affiliations:** 1 Department of Pediatrics, Tongji Hospital of Tongji Medical College, Huazhong University of Science and Technology, No. 1095 Jie Fang Avenue, Hankou, Wuhan, 430030 People’s Republic of China; 2 Department of Endocrinology, Shenzhen Children’s Hospital, Shenzhen, 518038 People’s Republic of China

**Keywords:** Isolated growth hormone deficiency, GH1, Gene, Mutation

## Abstract

**Background:**

A case of isolated growth hormone deficiency type IA (IGHD IA) caused by novel compound heterozygous mutation in the *GH1* gene was reported in this study, which aimed to provide insights that will benefit future diagnosis and treatment.

**Case presentation:**

We analyzed and summarized the clinical data and genetic test results from a patient with IGHD admitted in March 2019 to the Department of Pediatrics Tongji Hospital, Tongji Medical College, Huazhong University of Science and Technology. We described the results from a 1-year-9-months old female, whose chief complaint was “growth retardation for more than one year”. Her birth length was 49.0 cm, and her birth weight was 3.05 kg. Suboptimal intake (breastfeeding) jaundice lasted for approximately two months following birth. When evaluated at the age of 1-year-9-months old, the patient’s height was 61.0 cm (− 7.24 SD), and her weight was 6.4 kg (− 1.50 SD). The patient’s physical characteristics included yellowish hair, large and unclosed anterior fontanelles, raised forehead, and a low and flat nose. The major abnormalities observed from the auxiliary examinations included low GH (< 0.05 μg/l), low IGF-1 (16.99 μg/l), and elevated TSH (6.97 mIU/l). Genetic testing revealed two heterozygous variants: a splicing mutation (NG_011676.1(NM_022560.4): c.10 + 1G>T, inherited from her mother) in intron 1 of the *GH1* gene and a deletion that encompassed the same gene (chr17: 61973811–61996255, inherited from her father). After hormone replacement therapy with L-thyroxine and recombinant human GH (rhGH), the patient’s thyroid function returned to normal, and her serum IGF-1 level significantly improved, which resulted in an accelerated increase in height.

**Conclusion:**

This study described a case of IGHD caused by novel compound heterozygous mutations in the *GH1* gene. This study suggested that closer attention should be directed to genetic testing and diagnosis based on clinical characteristics to avoid misdiagnosis.

## Background

Isolated growth hormone deficiency (IGHD) is a common disease affecting the growth and development of children, with an incidence of 1/4000–1/10,000. IGHD can be classified as sporadic and familial, with the latter being rare (approximately 3% to 30%) [1]. IGHD can be subdivided further into three categories, IA (OMIM: 262400) and IB (OMIM:612781) (autosomal recessive inheritance), II(OMIM:173100) (autosomal dominant inheritance), and III (OMIM:307200) (X-linked inheritance) [2]. In 1970, Ruth Illig first reported three cases of IGHD IA, exhibiting growth hormone deficiency and severe growth retardation [3]. In 1972, Illig and Prader observed patients with IGHD IA who also presented distinctive facial features and a tendency to produce growth hormone antibodies that affected treatment in addition to the observed growth disorders. *GH1*(OMIM:139250) deletions resulting in IGHD IA have been reported, ranging in size from 6.5 to 45 kb. Deletions of 6.7 kb due to unequal recombination and crossover within the *GH* gene cluster during meiosis are the most common. In addition, heterozygous frameshift mutations and a homozygous nonsense mutation have been reported [4]. In patients with these mutations, severe growth retardation occurs in the first six months of life (height standard deviation score (HtSDS) less than − 4.50), GH is not detected, and the patients tend to produce GH antibodies that affect the therapeutic effects of exogenous rhGH. This study analyzed clinical data from a case of IGHD IA admitted to the Department of Pediatrics, Tongji Hospital, Tongji Medical College, Huazhong University of Science and Technology in March 2019 to improve the basic understanding of this disease.

## Case presentation

### Clinical data

#### General data

The proband, a 1-year-9-months old female, was admitted to the hospital in March 2019 for “growth retardation of more than one year”. She was born at term gestation to a G1P1(G is gravida, meaning the number of pregnancies; P is pregnancy, indicating the number of births) mother via cesarean section delivery. Her birth weight was 3.05 kg (25th–50th centile), and her birth height was 49.0 cm (25^th^–50th centile). She was breastfed to the age of one year, then fed with a combination of breastfeeding and formula. The child’s appetite was normal. However, she experienced postnatal jaundice when consuming breast milk that lasted for approximately two months. The child’s comprehension and memory were normal.

The child’s parents were from two unrelated families, and there was no family history of inherited or metabolic diseases. The heights of her father and mother were 171.0 cm and 167.0 cm, respectively. The body length of the child was monitored by her parents for the first nine months after birth, as seen in Table 1.

**Table 1:** Body length and SDS scores of the proband aged 0 to 9 months

Age (month)	0	2	4	6	9
Body length (cm)	49.0	51.0	54.0	57.0	58.0
Height SD score	− 0.41 SD	− 2.95 SD	− 4.10 SD	− 4.22 SD	− 5.14 SD

### Physical examination

The child’s height at the time of admission was 61.0 cm (− 7.24 SD), and her weight was 6.4 kg (− 1.50 SD). Specific physical characteristics included yellowish hair, large and unclosed anterior fontanelles, a raised forehead, a low and flat nose, and 12 erupted baby teeth. No apparent abnormalities were observed during cardiopulmonary auscultation. Her abdomen was soft, and her liver and spleen were palpable and exhibited normal size, smoothness, and firmness.

### Auxiliary examinations

No discernible abnormalities were found in the blood gas analysis, liver and kidney function assays, and serum electrolytes. The thyroid function indicators, including total triiodothyronine (TT3) (2.06 nmol/l), total thyroxine (TT4) (107.00 nmol/l), and free thyroxine (FT4) (1.15 ng/dl), were in the normal range. However, the TSH (6.97 mIU/l) level was elevated. The serum ACTH (29.80 pg/ml) and cortisol (414.16 nmol/l) levels were normal, but the IGF-1 (16.99 ng/ml) level was low, and the level of growth hormone was exceedingly low (< 0.05 μg/l). Fasting blood glucose (3.44 mmol/l) and insulin (0.16 mIU/l) levels also were low. The child's karyotype was determined to be 46, XX.

## Gene sequencing analysis

### Gene sequencing

After obtaining written, informed consent from her parents, whole-exome sequencing (WES) analysis and whole-genome copy number variation (CNV) detection (Beijing MyGenostics Inc.) were performed on the proband and her parents. A genome library was constructed using a standard library construction kit, and WES covered all exons of all genes (estimated to be approximately 20,000–25,000 genes) with a total number of captured exons estimated to be approximately 180,000 coding exons. The average coverage for all genes was greater than 97% at an average depth of 20X or higher. Suspected candidate mutations were screened by comprehensively considering the genetic pattern of the disease and the clinical characterization of the patient. Whole-genome CNV detection was performed by carrying out genome-wide CNV scanning on the samples, and the disease-free CNV region in the DGV database, specifically, the candidate CNV region, was filtered out.

### Genetic testing results

**Fig. 1:**
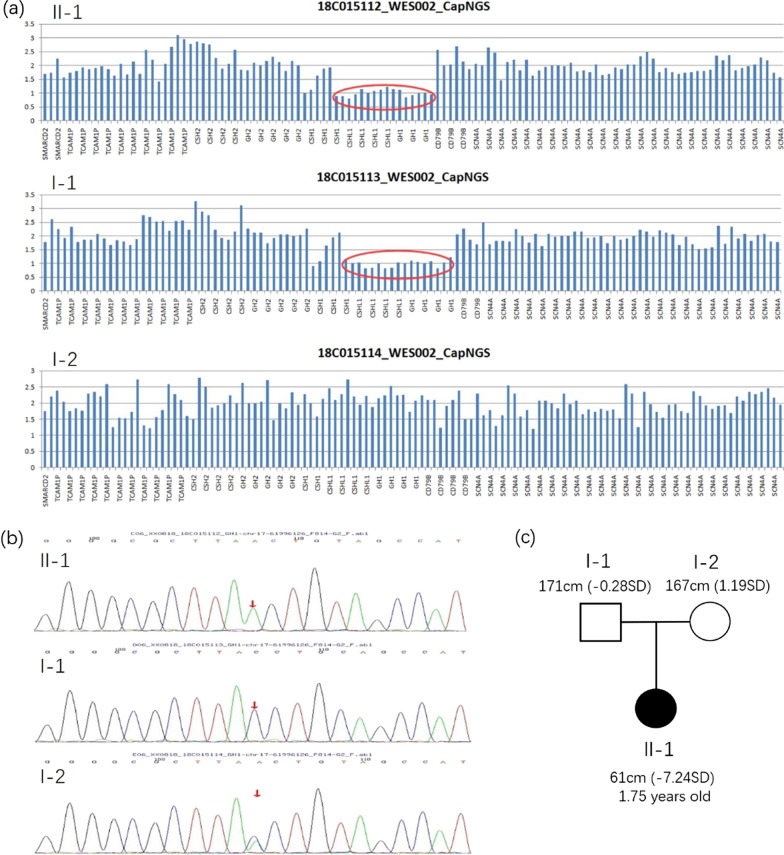
Whole-exome and Sanger sequencing results from the proband and her parents. **a** A deletion occurred in the detected region of chromosome 17 (chr17:61973811–61996255) of the proband (II-1) and the father (I-1). **b** A homozygous mutation (c.10 + 1G>T) existed in the proband (II-1), and a heterozygous mutation (c.10 + 1G>T) existed in the mother (I-2). **c** Pedigree of the proband (II-1), I-1 and I-2 represent the father and the mother

The genome-wide CNV detection found that the proband exhibited a 22 kb deletion in the chr17q23.3 region, suggesting that it might result in a pathogenic variant. The coverage of this genomic region by WES was decreased in both the proband and his father, suggesting that they might carry a heterozygous deletion encompassing this region (Fig. 1a). Moreover, a splicing mutation (NG_011676.1(NM_022560.4): c.10 + 1G>T) in the *GH1* gene intron 1 was inherited from her mother (Fig. 1b). The splicing mutation had not been reported previously. According to the 2019-ACMG Standards and Guidelines [5], the splicing mutation was a pathogenic variant due to the evidence of pathogenicity for PVS1 (nonsense, splicing variant), PP3 (harmful effects on genes or gene products), and PM2 (low-frequency variation in the normal population database).

### Diagnosis, treatment, and follow-up

**Fig. 2:**
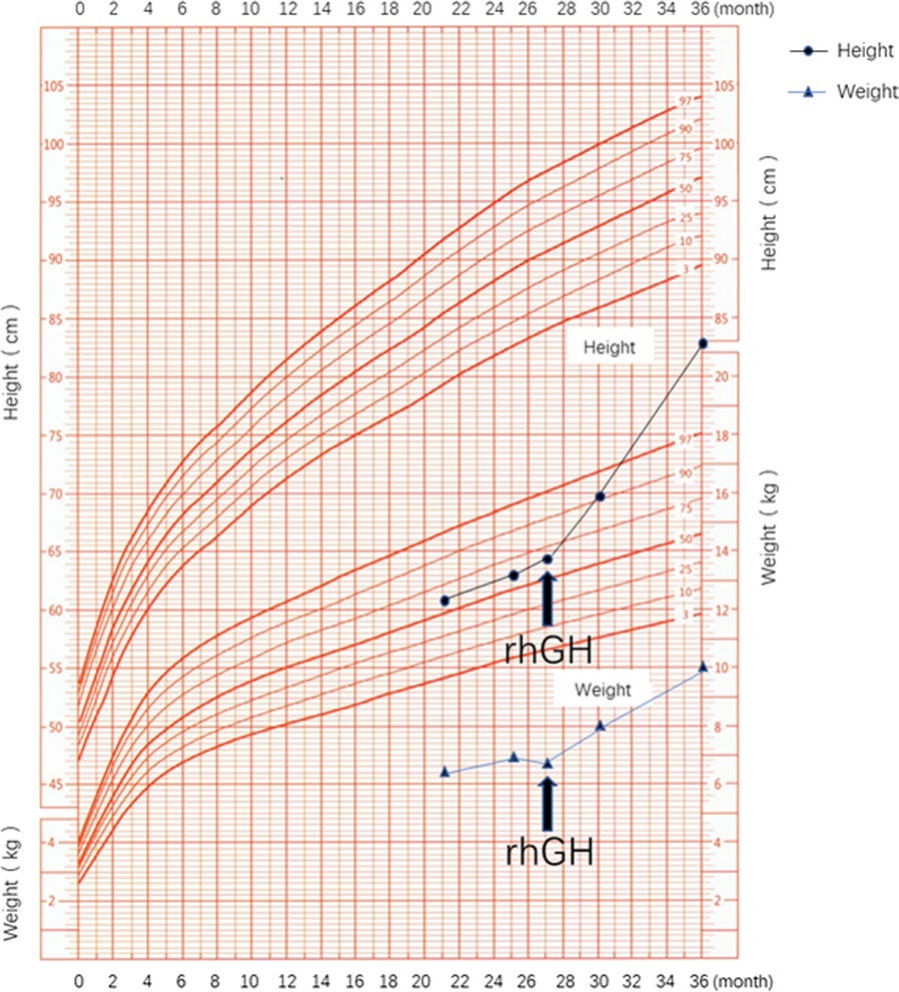
The figure shows the height and weight gain of the child before and after initiation of rhGH injections

After two weeks of treatment with L-thyroxine tablets, the child’s thyroid function returned to normal. The gene testing results indicated the suspect lesions. When these results were combined with the clinical characteristics of the affected child, we diagnosed IGHD IA caused by a *GH1* gene mutation. The child was treated with rhGH (0.03 mg/kg per day) via subcutaneous injection. After initiating the rhGH treatment, the child’s growth velocity (GV) and serum IGF-1 levels increased significantly. No adverse reactions were observed. The height and weight of the child that were monitored starting at the time of the voluntary diagnosis are shown in Fig. 2.

## Discussion and conclusions

IGHD Type IA is an autosomal recessive hereditary disease. It is clinically characterized by short stature accompanied by a decreased growth rate and severe growth retardation in the first six months of life (with a height SDS score less than -4.50 SD). The GH levels of patients with IGHD Type IA are undetectable, and anti-GH antibodies occur after exposure to rhGH [6]. Although the initial reaction to rhGH treatment might be favorable, there is a tendency to produce antibodies that adversely affect treatment [7]. Although children with IGHD exhibit normal body length and weight at birth, GHD caused by *GH1* gene mutations inhibits growth in body length after birth. Some patients may have small penises or exhibit fasting hypoglycemia. Other common characteristics reported in the literature include truncal obesity, a raised forehead, and a low and flat nasal bridge [8, 9]. Currently, most cases of IGHD reported in the literature are familial, and sporadic cases are rare. Therefore, IGHD should be considered as a differential diagnosis for children with severe growth retardation. Differential diagnosis was made on the basis of normal birth length and weight, growth retardation after birth, severe deficiency of growth hormone, combined with the results of gene mutation and pedigree analysis. For example, the children with Silver-Russel syndrome (RSS) (OMIM:180860) also showed short stature, but their growth hormone level were normal. Moreover, their birth length and weight were lower than those of children of the same gestational age due to intrauterine growth restriction, and they also had clinical characteristics such as postnatal feeding difficulties, special facial features, and limb asymmetry, which could be distinguished from IGHD IA. The need to improve early biochemical detection and genetic analysis is critical to provide a prompt etiological diagnosis, prevention, and treatment.

**Fig. 3:**
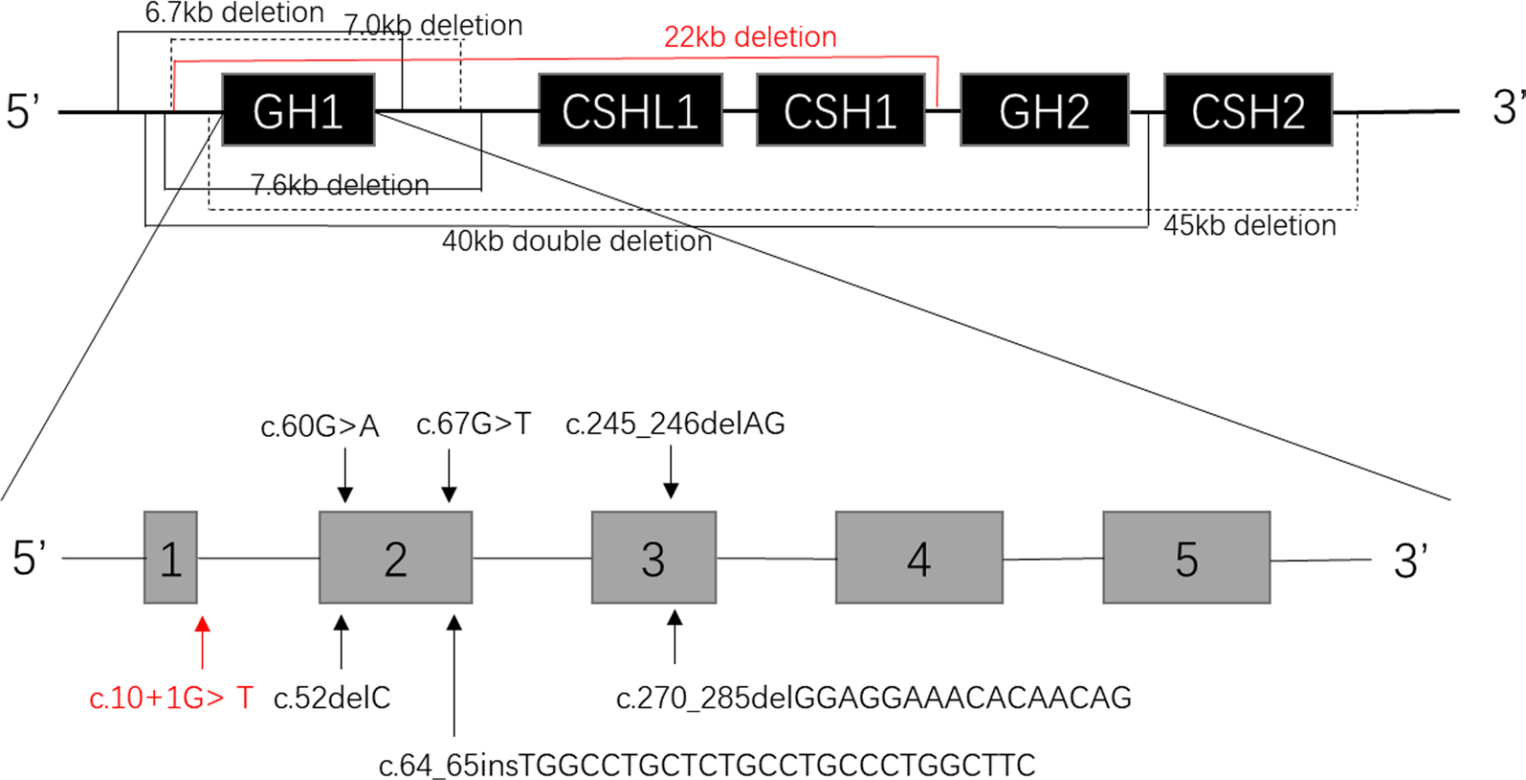
The schematic diagram shows five homologous gene clusters of the *GH1* gene and five exons of the *GH1* gene. These are the mutations that lead to IGHD IA, including deletions, coding region splicing mutations, point mutations, and insertions/deletions. The black font indicates information reported in the literature. The red font indicates the mutation type in this case

The facial and clinical features of the patient reported in this case were consistent with those reported in the literature for the most part. In this case, the patient exhibited severe growth retardation after birth until the initiation of treatment. Her growth hormone level (< 0.05 ng/ml) was extremely low, and her growth rate increased rapidly after initiating rhGH treatment. This patient exhibited a compound combination of two *GH1* gene mutations, including a splicing mutation in intron 1 inherited from her mother (NG_011676.1 (NM_022560.4): c.10 + 1G>T) and a missing fragment inherited from her father, which resulted in a *GH1* gene deletion. Based on the analysis of the family diagram, we speculated that both parents carried mutations. Therefore, two alleles, each with a mutation, were passed on to the child resulting in a compound heterozygous mutation of the *GH1* gene, which produced the phenotypic characteristics of IGHD Type IA (Fig. 1c). The gene mutations reported in the literature that cause IGHD Type IA are summarized and compared with this case, as seen in Fig. 3.

The most common genetic causes of IGHD include mutations in *GH1* and *GHRHR*. In addition, IGHD can be caused by mutations in transcription factors (*HESX1*,* SOX3*,* and OTX2*) or the development of a multi-pituitary hormone deficiency [10]. The *GH1* gene is located on the long arm of chromosome 17 (17q22-24). It is composed of five homologous genes, which are, in sequence, from 5′ to 3′, *GH1*, *CSHL1*, *CSH1*, *GH2,* and *CSH2*, and spans a region that is approximately 65 KD [11]. This gene cluster is consistent with respect to the coding region, insertion sequence, and flanking sequence. These homologous sequences appear upstream and downstream of the *GH1* gene, providing the basis for the unequal recombination of the *GH1* gene, which results in the deletion of the gene. Several published reports have described deletions in the *GH1* gene of different lengths, including 6.7 kb, 7.7 kb, 7.6 kb, 45 kb, and double deletions, that all resulted in IGHD IA. Of these reported deletions, the 6.7 kb deletion was the most common (80%). Heterozygous frameshift variants and a homozygous nonsense mutation also have been reported [4].

IGHD can be divided into four types, including autosomal recessive (IA and IB), autosomal dominant (II), and X-linked (III). The phenotypic characteristics of IGHD IA have been described above. IGHD IB is primarily caused by *GH1* or *GHRHR* mutations. Compared with IGHD IA, patients with IGHD IB exhibit more variable phenotypes. Children exhibit short stature, a slow growth rate, delayed bone age, low but detectable growth hormone concentrations, and respond favorably to exogenous growth hormone treatment [12, 13]. IGHD II is the most common genetic form of IGHD, and children show considerable variability in the time of onset and severity of GHD. Over time, they might develop deficiencies in other pituitary hormones [14, 15], requiring lifelong follow-up assessments. IGHD III can be caused by mutations in *SOX3* or *BTK* genes [16], and patients often exhibit mental retardation, craniofacial malformations, hypoglycemia, and agammaglobulinemia [17–19]. The phenotypic characteristics of each genetic form of IGHD are similar, but they also exhibit specific phenotypic characteristics, which should be distinguished through clinical assessments. In addition, *GH1* gene mutations can cause IGHD IB, IGHD II, and Kowarski syndrome. Patients with these conditions exhibit exceedingly short stature, although their specific clinical phenotypes are not identical because of the type and site of the mutations. Overall, the *GH1* genotype is closely associated with the phenotype of short stature caused by the *GH1* mutations.

Despite the increasing number of genes known to be involved in IGHD etiology, mutations in known genes account for only a small number of cases. Therefore, understanding emerging, newly discovered mutations can guide clinical diagnosis and facilitate patient treatment. Early application of rhGH to treat IGHD IA has demonstrated favorable results. However, antibodies to rhGH eventually are produced during treatment. Therefore, long-term follow-up assessments must be conducted. Also, the presence of circulating growth hormone antibodies will substantially influence the ongoing treatment of these children, making it difficult for them to reach their expected height. Thus, further research is needed to improve the prognosis for the affected children.

## Data Availability

The *GH1* variant can be found in NCBI Nucleotide under the accession number NM_022560. The raw datasets generated and analyzed during the current study are not publicly available in order to protect participant confidentiality. The datasets analyzed during the current study are available from the corresponding author on reasonable request.

## Abbreviations

IGHD: Isolated growth hormone deficiency
CNV: Copy number variation
GH: Growth hormone
IGF-1: Insulin-like growth factor-1
TSH: Thyroid stimulating hormone
rhGH: Recombinant human growth hormone
HtSDS: Height standard deviation score
TT3: Total triiodothyronine
TT4: Total thyroxine
FT4: Free thyroxine
ACTH: Adrenocorticotropic hormone
ACMG: American College of Medical Genetics
GHRHR: Growth hormone releasing hormone receptor
GV: Growth velocity
CSHL1: Chorionic somatomcmotropin hormone like 1
CSH1: Chorionic somatomammotropin hormone 1
GH2: Growth hormone 2
CSH2: Chorionic somatomammotropin hormone 2
HESX1: HESX homeobox 1
SOX3: SRY-box transcription factor 3
OTX2: Orthodenticle homeobox 2

## References

[1] Mullis PE (2007). Genetics of growth hormone deficiency. Endocrinol Metab Clin North Am.

[2] Procter AM (1998). The molecular genetics of growth hormone deficiency. Hum Genet.

[3] Illig R (1970). Growth hormone antibodies in patients treated with different preparations of human growth hormone (HGH). J Clin Endocrinol Metab.

[4] Wagner JK (1998). Prevalence of human GH-1 gene alterations in patients with isolated growth hormone deficiency. Pediatr Res.

[5] Brandt T (2020). Adapting ACMG/AMP sequence variant classification guidelines for single-gene copy number variants. Genet Med.

[6] Pinto G (1997). Pituitary stalk interruption syndrome: a clinical-biological-genetic assessment of its pathogenesis. J Clin Endocrinol Metab.

[7] Alatzoglou KS (2014). Isolated growth hormone deficiency (GHD) in childhood and adolescence: recent advances. Endocr Rev.

[8] Ioimo I (2018). Same phenotype in children with growth hormone deficiency and resistance. Case Rep Pediatr.

[9] Keselman A (2012). Type IA isolated growth hormone deficiency (IGHD) consistent with compound heterozygous deletions of 6.7 and 7.6 Kb at the GH1 gene locus. Arq Bras Endocrinol Metabol.

[10] Alatzoglou KS, Dattani MT (2010). Genetic causes and treatment of isolated growth hormone deficiency-an update. Nat Rev Endocrinol.

[11] Alatzoglou KS, Dattani MT (2012). Phenotype-genotype correlations in congenital isolated growth hormone deficiency (IGHD). Indian J Pediatr.

[12] Cogan JD (1993). Heterogeneous growth hormone (GH) gene mutations in familial GH deficiency. J Clin Endocrinol Metab.

[13] Iughetti L (2008). Complex disease phenotype revealed by GH deficiency associated with a novel and unusual defect in the GH-1 gene. Clin Endocrinol (Oxf).

[14] Mullis PE (2005). Isolated autosomal dominant growth hormone deficiency: an evolving pituitary deficit? A multicenter follow-up study. J Clin Endocrinol Metab.

[15] Turton JP (2006). Evolution of gonadotropin deficiency in a patient with type II autosomal dominant GH deficiency. Eur J Endocrinol.

[16] Stewart DM (2008). X-linked hypogammaglobulinemia and isolated growth hormone deficiency: an update. Immunol Res.

[17] Alatzoglou KS (2011). Increased transactivation associated with SOX3 polyalanine tract deletion in a patient with hypopituitarism. J Clin Endocrinol Metab.

[18] Laumonnier F (2002). Transcription factor SOX3 is involved in X-linked mental retardation with growth hormone deficiency. Am J Hum Genet.

[19] Solomon NM (2004). Array comparative genomic hybridisation analysis of boys with X linked hypopituitarism identifies a 3.9 Mb duplicated critical region at Xq27 containing SOX3. J Med Genet.

